# TREM‐1 deficiency attenuates the inflammatory responses in LPS‐induced murine endometritis

**DOI:** 10.1111/1751-7915.13467

**Published:** 2019-07-31

**Authors:** Hongmei Zhu, Wenke Li, Zhuole Wang, Jianguo Chen, Mingxing Ding, Li Han

**Affiliations:** ^1^ College of Veterinary Medicine Huazhong Agricultural University Wuhan 430070 China

## Abstract

Endometritis, which is usually caused by bacterial infection, is characterized by high levels of pro‐inflammatory cytokines and a high infertility rate. Triggering receptor expressed on myeloid cells‐1 (TREM‐1) has been recognized as a potent amplifier of inflammatory reactions. Studies have demonstrated reduced inflammatory responses and mortality rates of animals with bacterial infection due to the blocking of TREM‐1 expression. However, whether TREM‐1 deficiency could alleviate the inflammatory reaction in bacterial endometritis is still unclear. Here, TREM‐1 knock‐out (*Trem‐1^−/−^*) mice were used to inhibit TREM‐1 signalling to evaluate its role in inflammatory reactions after a highly pathogenic LPS infection in mice uteri. The results demonstrated that TREM‐1 deficiency attenuated the inflammation in mice uteri; markedly reduced the number of polymorphonuclear neutrophils; and suppressed interleukin‐1β (IL‐1β), IL‐6, and tumour necrosis factor‐α (TNF‐α) concentrations in serum as well as their production in inflamed uteri after LPS stimulation. Our results illustrate an anticipated pathogenic impact of TREM‐1 on endometritis during LPS infection and indicate that blocking of TREM‐1 in LPS‐induced endometritis holds considerable promise for blunting excessive inflammation.

## Introduction

Postpartum uterine disease in animals, which is usually caused by persisting bacterial contamination of the uterine lumen, is the major cause of infertility in cows, mares and sows (Albihn, *et al.*, 2003; Sheldon and Dobson, 2004; Sheldon, *et al.*, 2006; Jana, *et al.*, 2014). The most commonly occurring postpartum uterine diseases in domestic animals can be classified as metritis, endometritis and pyometra (Abiven, *et al.*, 1998; Sheldon, *et al.*, 2006; Katila, 2016). Endometritis is one of the major postpartum uterine diseases of females and is considered as the leading cause of infertility and economic loss in the cattle, equine and swine breeding industries (Beutler, *et al.*, 2003; Sheldon, *et al.*, 2006; LeBlanc and Causey, 2009; Canisso, *et al.*, 2016; Wang, *et al.*, 2017). At present, endometritis can be categorized as clinical and subclinical endometritis. In cows, for example clinical endometritis is characterized by the detectable presence of purulent or mucopurulent (> 50% pus or approximately 50% mucus respectively) in uterine discharge in the vagina 21 or 26 days after parturition; the cows with clinical endometritis show the presence of chronic and/or acute endometrial inflammation on endometrial biopsies (LeBlanc, *et al.*, 2002; Sheldon, *et al.*, 2006). In the absence of characteristics in clinical endometritis, a cow with subclinical endometritis is defined by > 18% or > 10% neutrophils in uterine cytology samples collected 21–33 or 34–47 days postpartum; subclinical animals are clinically healthy but may have a relatively reduced pregnancy rate (Kasimanickam, *et al.*, 2004; Sheldon, *et al.*, 2006). In horses, endometritis often occurs in mares that fail to clear the inflammation within 36–72 h after mating or infection by microorganisms; clinical endometritis shows the signs of accumulation of intrauterine fluid, vaginitis, vaginal discharge, short interoestrous intervals and neutrophilic uterine cytology, while subclinical endometritis may only present with polymorphonuclear neutrophils (PMNs) in cytology with subacute inflammation but no bacteria (Albihn, *et al.*, 2003; Katila, 2016; Troedsson and Woodward, 2016). Equine endometritis was rated as the third most common problem in adult mares in North America, and the pregnancy rates of mares experiencing severe endometritis were reported to be as low as 21% (Traub‐Dargatz, *et al.*, 1991; Riddle, *et al.*, 2007). For sows, it has also been reported that infectious endometritis is a major challenge in sow reproduction (Jana, *et al.*, 2014; Jana, *et al.*, 2015).

At the cellular and molecular level, an animal with endometritis always shows increased levels of PMNs; high endometrial expression of pro‐inflammatory cytokines or mediators, such as interleukin 1β (IL‐1β), IL‐6, IL‐8, tumour necrosis factor‐α (TNF‐α); antimicrobial peptides; acute phase proteins; and prostaglandins (Mateus, *et al.*, 2003; Kasimanickam, *et al.*, 2004; Fischer, *et al.*, 2010; Christoffersen, *et al.*, 2012). Generally, the Toll‐like receptor 2 (TLR2) or TLR4, which mediate the inflammatory reactions, has also been noted in infertile animals with a history of endometritis (Siemieniuch, *et al.*, 2016; Schoniger, *et al.*, 2017; Yin, *et al.*, 2019). Studies have reported that a wide variety of bacterial species is present in the uterine lumen of dairy cows during the early postpartum period; *Escherichia coli* (*E. coli*), *Trueperella pyogenes*, *Streptococcus spp.*, *Staphylococcus spp.*, *Pseudomonas spp*., *Clostridium spp*. and various Gram‐negative anaerobic species are regarded as the most dominant pathogens in the bovine uterus (Bicalho, *et al.*, 2012; Sens and Heuwieser, 2013). Some of these bacterial species can persist in the uterine lumen and lead to the development of postpartum metritis and endometritis. It has been suggested that Gram‐negative *E. coli* is the most prevalent pathogen in the uterine lumens of dams (Bicalho, *et al.*, 2012). Lipopolysaccharide (LPS) is the endotoxin found in the cell walls of Gram‐negative bacteria and plays an important role in the development of infectious disease. Known for its strong biological activities of entering the bloodstream and promoting the release of pro‐inflammatory mediators such as TNF‐α and IL‐1β, LPS has been widely used as a mimic of Gram‐negative pathogens such as *E. coli*, to establish animal models of inflammatory disease, including endometritis (Lv, *et al.*, 2015; Jiang, *et al.*, 2018).

Triggering receptor expressed on myeloma cells‐1 (TREM‐1) is a member of the immunoglobulin superfamily and is expressed on the cell surfaces of neutrophils and certain monocytes that infiltrate inflamed tissues (Bouchon, *et al.*, 2000; Bouchon, *et al.*, 2001). It has been verified over decades that TREM‐1 can activate neutrophils and monocytes through the transmembrane adapter protein DAP12 and amplify inflammatory responses to microbial products. Regarding this critical function, TREM‐1 is considered an attractive target for the treatment of sepsis and inflammatory disease (Jung, *et al.*, 2012; Horst, *et al.*, 2013). In addition, TREM‐1 expression is strongly upregulated by extracellular bacteria or their microbial products, such as *Pseudomonas aeruginosa*, *Staphylococcus aureus*, lipoteichoic acid (LTA) or LPS (Bouchon, *et al.*, 2001; Cohen, 2001). The mechanism for TREM‐1 increasing LPS‐induced inflammation is that TREM‐1 can synergize with LPS to induce the secretion of pro‐inflammatory cytokines (e.g. TNF‐α, IL‐1β, IL‐8 and IL‐6) or to induce the release of myeloperoxidase (MPO) (Bouchon, *et al.*, 2001). Furthermore, inhibition of TREM‐1 either by antibodies or by gene targeting could protect mice against LPS or live *E. coli*‐induced shock or sepsis (Bouchon, *et al.*, 2001; Liu, *et al.*, 2016). Additionally, it has been demonstrated that TREM‐1‐Ig Fc fusion protein treatment could lower the serum levels of TNF‐α and IL‐1β after LPS administration (Wang, *et al.*, 2012). These results demonstrate that TREM‐1 is a potential therapeutic target because of its critical function in inflammatory responses to bacteria and LPS.

Given that TREM‐1 plays a vital role in inflammatory responses to bacteria, we want to investigate whether TREM‐1 is involved in the inflammatory process in LPS‐induced endometritis and whether genetic deletion of TREM‐1 could alleviate the inflammatory reaction after *E. coli‐*derived LPS infection in the uterus. These findings would not only demonstrate an unanticipated clear role for TREM‐1 in LPS‐induced endometritis, but also illustrate the potential for a novel therapeutic intervention in infectious endometritis.

## Results

### Effect of LPS on morphological changes of the uteri in *Trem‐1^−/−^* mice

**Figure 1:**
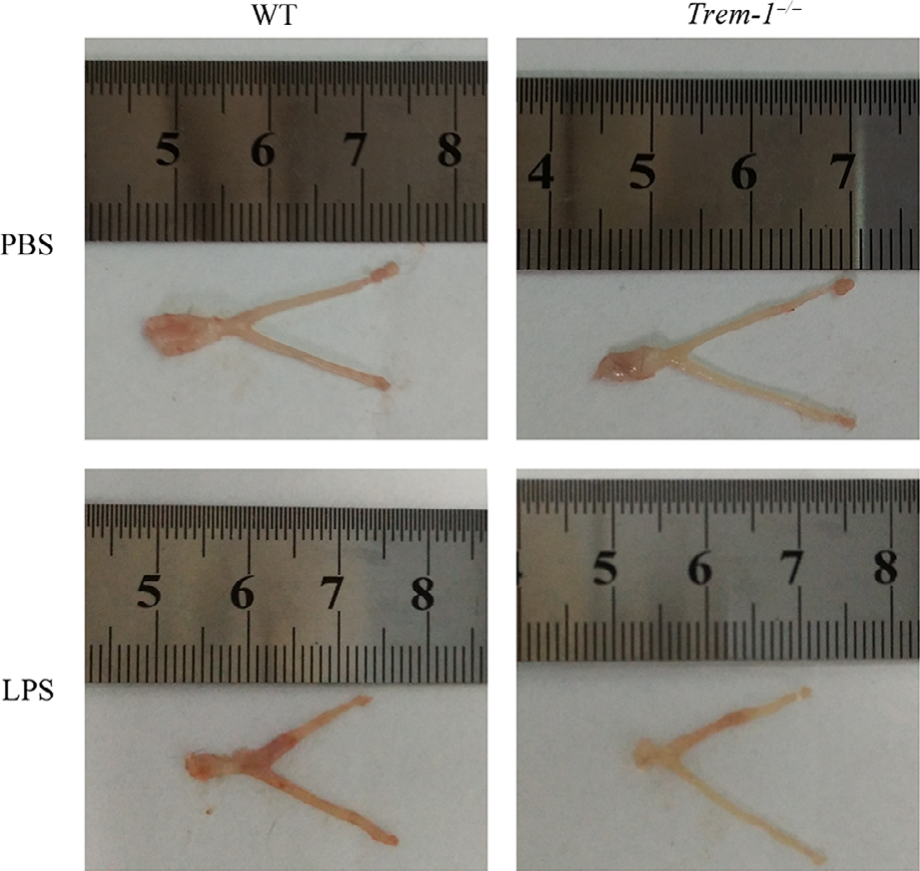
Morphological changes in the uterus of TREM‐1 deficient mice after LPS treatment. The images show the morphological changes of the uteri of WT and *Trem1^−/−^* mice after PBS or LPS treatment.

In the current study, an LPS‐induced endometritis mouse model was created to determine whether *Trem‐1* knockout affected the inflammatory responses of the uterus. Wild‐type C57BL/6 (WT) mice were used as experimental controls for the TREM‐1 knockout C57BL/6 (*Trem‐1^−/−^*) mice. The WT and *Trem‐1^−/−^* mice were all given phosphate buffer saline (PBS) and LPS by uterine perfusion. The PBS‐treated mice were used as negative controls for the LPS‐treated mice. As shown in Fig. 1, oedema and haemorrhages were found in the uteri of mice after LPS treatment compared with the mice receiving PBS. Nevertheless, the damage to uteri in the WT group mice was more severe than that in the *Trem‐1^−/−^* group mice after LPS treatment. This demonstrates that the destruction of the uterine structure in the *Trem‐1^−/−^* group was significantly relieved in comparison with the WT group and that the TREM‐1 deficiency protects mice from LPS‐induced inflammatory injury of the uterus.

### **Effect of LPS on histopathological changes of the uteri in *Trem‐1*** *^−/−^* **mice**

**Figure 2:**
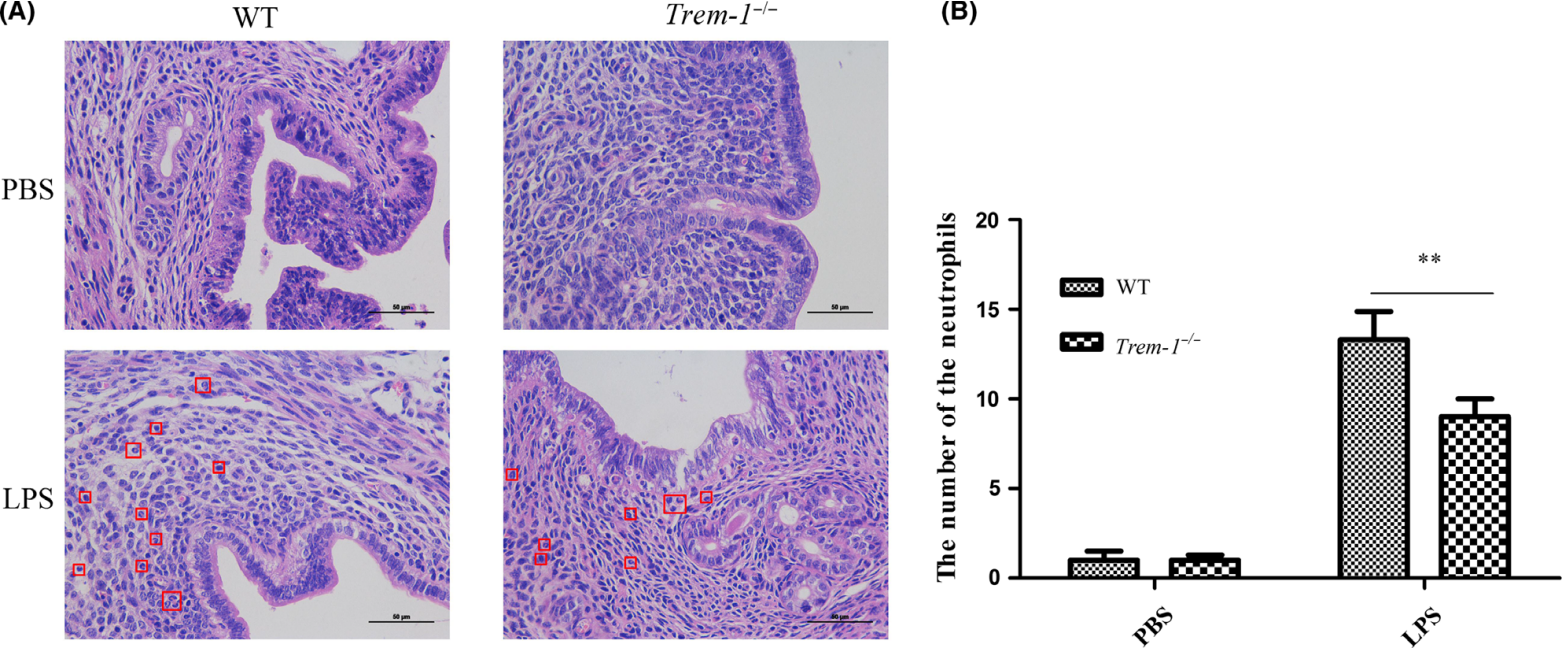
Effect of TREM‐1 deficiency on histopathological changes in the uterus after LPS processes. A. The uterine histopathological changes of mice in each group after PBS and LPS process. The red box represents PMNs. B. The mean number of neutrophils was assessed from at least three replicates of the samples. Student’s *t*‐test was performed for determining the significance. Data are shown as the means ± SD. **P < 0.01.

To further investigate the uterine changes after LPS treatment, we performed H&E staining on uteri from different groups. Figure 2A shows that the uteri of the mice receiving LPS perfusion were significantly damaged and were surrounded and infiltrated with PMNs. Furthermore, neutrophil counts were lower in the *Trem‐1^−/−^* group than in the WT group after LPS treatment (*P* < 0.01) (Fig. 2B).

### **Effect of LPS on the serum levels of pro‐inflammatory cytokine in *Trem‐1*** *^−/−^* **mice**

**Figure 3:**
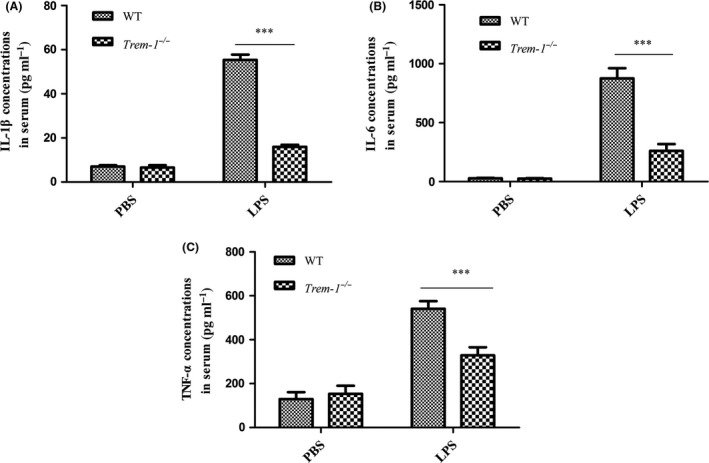
Effect of TREM‐1 deficiency on pro‐inflammatory cytokine production in the serum after LPS stimulation. A. IL‐1β concentrations. B. IL‐6 concentrations. C. TNF‐α concentrations. The levels of the cytokines were detected from at least three replicates of the samples by ELISA assay. Student’s *t*‐test was performed for determining the significance. Data are shown as the means ± SD. ***P < 0.001.

The serum levels of the pro‐inflammatory cytokines of IL‐1β, IL‐6 and TNF‐α were measured to further evaluate the inflammatory responses. The results revealed that these cytokines were significantly upregulated in the LPS‐treated group mice compared with those in the PBS‐treated mice (IL‐1β: LPS‐treated vs. PBS‐treated, *P* < 0.001; IL‐6: LPS‐treated vs. PBS‐treated, *P* < 0.01; TNF‐α: LPS‐treated vs. PBS‐treated, *P* < 0.01) (Fig. 3). These results further demonstrated the severe inflammatory reaction after LPS treatment. However, after LPS perfusion, serum IL‐1β (Fig. 3A), IL‐6 (Fig. 3B) and TNF‐α (Fig. 3C) concentrations in the *Trem‐1^−/−^* mice were greatly reduced when compared with those in WT mice (*P* < 0.001, *P* < 0.001 and *P* < 0.001 respectively).

### **Effect of LPS on the pro‐inflammatory cytokine expression in the uteri of *Trem‐1*** *^−/−^* **mice**

**Figure 4:**
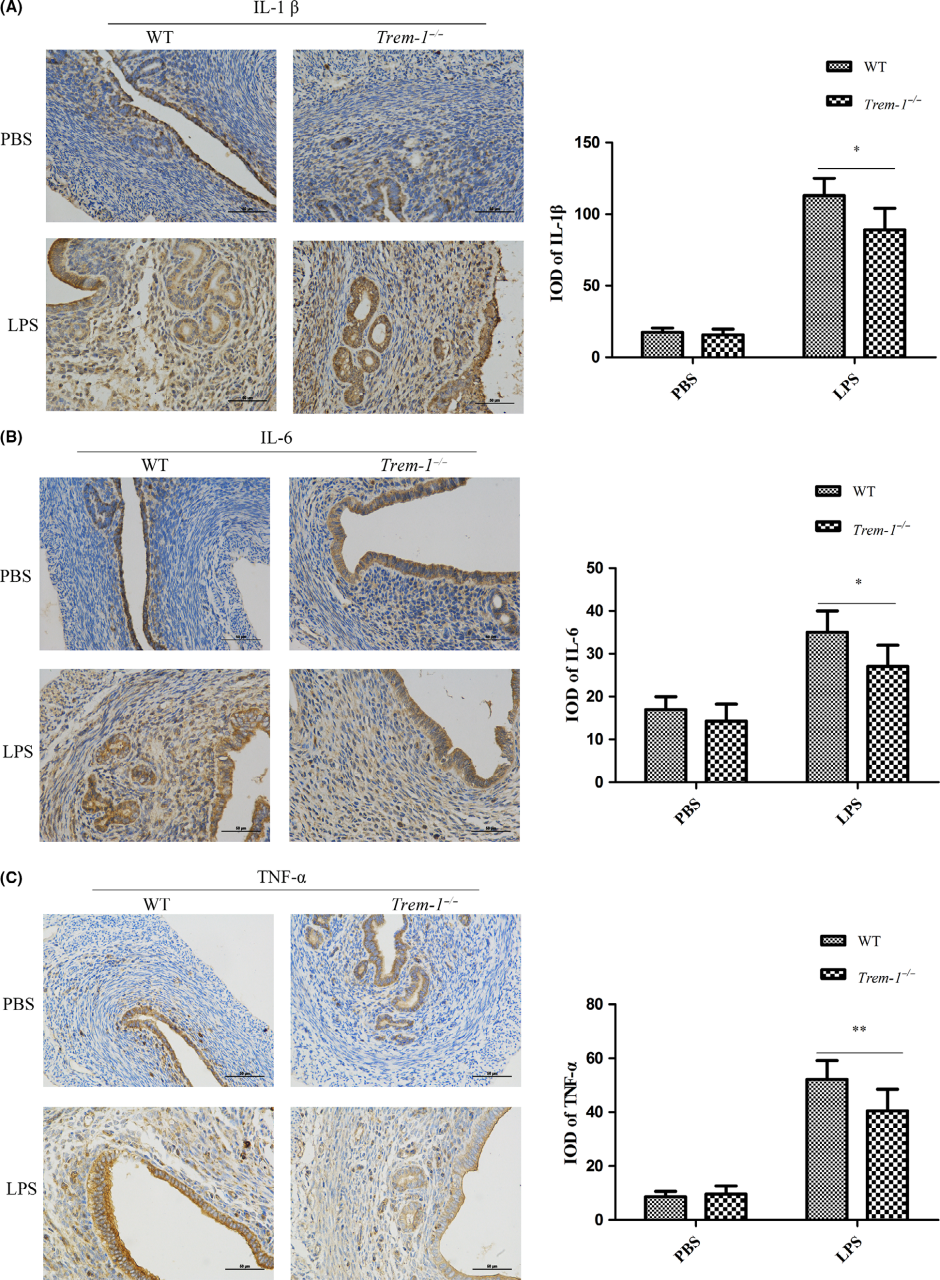
Effects of TREM‐1 deficiency on pro‐inflammatory cytokines expressions in uteri after LPS stimulation. A. IL‐1β expression in uteri (Left) and the IOD analysis (Right). B. IL‐6 expression in uteri (Left) and the IOD analysis (Right). C. TNF‐α expression in uteri (Left) and the IOD analysis (Right). The levels of the immunoreactive cells for IL‐1β, IL‐6 and TNF‐α were detected by integrated optical density (IOD) from at least three replicates. Student’s t‐test was performed for determining the significance. Data are shown as the means ± SD. *P < 0.05, **P < 0.01.

To investigate inflammatory cytokine expression in the uterus, we performed an immunohistochemical analysis of IL‐1β (Fig. 4A), IL‐6 (Fig. 4B) and TNF‐α (Fig. 4C) expression in inflamed uteri. The results demonstrated that there were no differences in the integrated optical density (IOD) of immunoreactive cells for IL‐1β, IL‐6 or TNF‐α expression after PBS treatment. However, compared with the PBS‐treated mice, the IOD of immunoreactive cells for IL‐1β, IL‐6 and TNF‐α in uteri was greatly increased in the LPS‐treated groups (IL‐1β: LPS‐treated vs. PBS‐treated, *P* < 0.01; IL‐6: LPS‐treated vs. PBS‐treated, *P* < 0.05; TNF‐α: LPS‐treated vs. PBS‐treated, *P* < 0.01). Nevertheless, the IOD of immunoreactive cells for IL‐1β, IL‐6 and TNF‐α was lower in the *Trem‐1^−/−^* mice than in the WT mice after LPS treatment (*P* < 0.05, *P* < 0.05 and *P* < 0.01 respectively).

## Discussion

Endometritis has been drawing increasing attention for its impairment of the reproductive performance of dams and reduction in breeding industry profits (Gilbert, *et al.*, 2005; Lee and Kim, 2007; McDougall, *et al.*, 2011). To investigate the bacterial endometritis, several models have been established in mice or livestock in previous reports (Hinrichs, *et al.*, 1992; Lv, *et al.*, 2015; Shao, *et al.*, 2017; Ferris, *et al.*, 2017; Piersanti, *et al.*, 2019). Among these models, LPS has been demonstrated to be an efficient tool to establish the mouse bacterial endometritis model (Jiang, *et al.*, 2018; Wang, *et al.*, 2019). The LPS‐induced endometritis mouse model showed numerous assembled PMNs, abundant secretion of inflammatory cytokines and the histopathological features in the inflammatory tissue that is regarded as the criteria to determine the obvious inflammatory changes of the uterus (Matteo, *et al.*, 2009). In the present study, we found that the uteri of LPS‐treated mice were more severely damaged and were infiltrated with large numbers of PMNs, illustrating the success of the model. However, the less severely damaged uteri and the lower numbers of PMNs in *Trem‐1^−/−^* mice than in the WT mice indicated that *Trem‐1* deficiency protected the mice from the LPS‐induced uterine injury and reduced the LPS‐induced recruitment of granulocytes to the inflamed tissues.

However, evidence suggests that TREM‐1 plays an essential role in modulating the innate immune response and amplifying the non‐infectious or infectious inflammation (Weber, *et al.*, 2014). Thus, numerous studies have been performed to further elucidate the role of TREM‐1 in sepsis or inflammatory disease by blocking or enhancing the TREM‐1 function (Yang, *et al.*, 2015; Tang and Dong, 2017). Studies addressing the impact of TREM‐1 on disease have so far mostly relied on the use of TREM‐1/Ig fusion proteins or synthetic peptides mimicking part of the extracellular domain of TREM‐1 to block TREM‐1 signalling (Tammaro, *et al.*, 2016; Rao, *et al.*, 2019). Although use of these agents has been shown to provide substantial protection from disease, the prevention of additional potential receptors and the controversial findings from impairing TREM‐1 prompted us to choose a more efficient method to block TREM‐1 signalling. *Trem‐1^−/−^* mice have been widely used for investigating the precise and the true biological roles of TREM‐1 in inflammatory diseases (Weber, *et al.*, 2014; Joffre, *et al.*, 2016; Kökten, *et al.*, 2017). We thus exploited the *Trem‐1^−/−^* mice to inhibit the TREM‐1 function in the current study. Our results demonstrate that genetic deletion of the TREM‐1 gene greatly reduced the inflammatory responses of mice experiencing the LPS stimulation, which was consistent with the effects of TREM‐1 inhibition on sepsis or septic shock induced by *E.coli*, *Streptococcus pyogenes* or LPS (Bouchon, *et al.*, 2001).

*Escherichia coli‐*derived LPS can promote a TLR4‐dependent inflammatory response, leading to the production of pro‐inflammatory cytokines, such as IL‐1β, IL‐6 and TNF‐α (Chow, *et al.*, 1999; Beutler, *et al.*, 2003; Akira and Takeda, 2004). These cytokines are major pro‐inflammatory cytokines that directly reflect the measures of innate immune cells activity, such as numbers of neutrophils and monocytes in tissue. In addition, IL‐1β plays a vital role in the synthesis of prostaglandin and fibrinogen (Weinstein and Taylor, 1987). IL‐6 exhibits a profibrotic effect by supporting the survival of Th‐1 cells and upregulating IFN‐γ production, which in turn downregulates matrix metalloproteinase activity (Zhang, *et al.*, 2012; Fielding, *et al.*, 2014; O'Reilly, *et al.*, 2014). TNF‐α is reported to be highly virulent to the uterus and could induce the apoptosis of endometrial epithelial cells. Suppressing the expression of TNF‐α could attenuate LPS‐induced pro‐inflammatory cytokine secretion in macrophages (Pallai, *et al.*, 2016). In the present study, the higher expressions of IL‐1β, IL‐6 and TNF‐α in serum or inflammatory endometrium after LPS treatment suggested an acute inflammatory reaction and potential fibrinogen and cell apoptosis in the uterus. Previous studies reported that inhibition of TREM‐1 suppressed IL‐1β‐induced chondrocyte injury, reduced colitis in mice, alleviated *Streptococcus*‐induced syndrome and attenuated LPS‐induced acute lung injury (Liu, *et al.*, 2016; Kökten, *et al.*, 2017; Tang and Dong, 2017; Han, *et al.*, 2018). Consistent with these results, the lower levels of IL‐1β, IL‐6 and TNF‐α in serum or uteri in the *Trem1^−/−^* mice than in the wild‐type mice after LPS stimulation suggested that the TREM‐1 gene knockout could attenuate LPS‐induced endometritis, probably by inhibiting inflammatory cytokines.

In summary, the present study provided strong evidence of the protective effects of TREM‐1 deficiency against LPS‐induced endometritis in mice, the mechanism of which could be deduced as knocking out of the *Trem‐1* inhibited the LPS‐induced production of IL‐1β, IL‐6 and TNF‐α. The results of the present study suggest the potential of TREM‐1 deficiency for the treatment of *E. coli‐*infected endometritis.

## Experimental procedures

### Animals and ethics statement

A total of twenty‐four 6‐week‐old female C57BL/6 WT and *Trem‐1^−/−^* mice with similar body weights were obtained from the Experimental Animal Center of Huazhong Agricultural University (Wuhan, China). The mice were maintained in a temperature‐controlled housing with a 12 h light/12 h dark cycle and were provided with food and water ad libitum for at least 1 week before the experiment. The animal experiments in this study were performed in strict accordance with the Guide for the Care and Use of Laboratory Animals of the Monitoring Committee of Hubei Province, China. The protocol was approved by the Committee on the Ethics of Animal Experiments at the College of Veterinary Medicine, Huazhong Agricultural University. All efforts were made to minimize the suffering of the mice.

### Mouse model of endometritis

In the experiments, all mice were determined to be in dioestrus. The LPS derived from the *E. coli* 055:B5 (Sigma‐Aldrich, St. Louis, MO, USA) was used to establish the endometritis model which was performed in accordance with the previous reports (Lv, *et al.*, 2015; Wang, *et al.*, 2019). Briefly, four groups of animals were constituted for establishing the endometritis model: (i) WT mice were given 20 μl PBS by uterine perfusion (*n* = 7). (ii) *Trem‐1^−/−^* mice were given 20 μl PBS by uterine perfusion (*n* = 5). (iii) WT mice were given 20 μl LPS (2.5 mg ml^−1^) by uterine perfusion (*n* = 7). (IV) *Trem‐1^−/−^* mice were given 20 μl LPS (2.5 mg ml^−1^) by uterine perfusion (*n* = 5). The first two groups receiving PBS treatment were used as controls for the WT or *Trem‐1^−/−^* mice receiving LPS management respectively. After 24 h, the blood and the middle part of the uterine horn of each mouse were collected after the experiments.

### Histological assay

The uterine tissues were harvested and immersed in 4% neutral polyformaldehyde for 48 h. The tissues were then embedded in paraffin, cut into 5‐μm sections and stained with haematoxylin and eosin (H&E). The stained tissues were examined under a microscope (Olympus, Tokyo, Japan). The histological examination was performed in a blind fashion; a combined analysis of tissue damage and inflammatory cell infiltration was conducted. The observation of uterine morphology, oedema, haemorrhage and pathological changes with the increased infiltration of PMNs can reflect the inflammation of the uterus, according to previous studies (Lv, *et al.*, 2015; Yin, *et al.*, 2019).

### ELISA

Blood samples were collected to acquire the serum. The concentrations of IL‐1β, IL‐6 and TNF‐α were measured with enzyme‐linked immunosorbent assay (ELISA) in accordance with the manufacturer’s protocols for IL‐1β, IL‐6 and TNF‐α kits respectively (Dakewe Biotech Co., Ltd., Shenzhen, China). The sensitivity of the assay was 22 pg ml^−1^ for IL‐1β, 7 pg ml^−1^ for IL‐6 and 8 pg ml^−1^ for TNF‐α. Intra‐ and interassay coefficients of variation were < 10% and < 15% respectively, for IL‐1β, IL‐6 and TNF‐α.

### Immunohistochemistry

Uterine tissues were fixed with 4% neutral polyformaldehyde for 48 h. The tissues were then embedded in paraffin and cut into 5‐μm sections. Immunohistochemical staining was performed according to the SABC immunohistochemical staining kit for the rabbit IgG (SA1022; Boster, Wuhan, China). Briefly, the paraffin sections were dewaxed to water routinely and then were incubated in 3% H_2_O_2_ deionized water to eliminate the endogenous peroxidase activity. The sections were incubated in the 95°C citrate buffer for 20 min to repair the antigen. After blocking in the 5% BSA for 30 min, the sections were incubated with the primary antibodies anti‐TNF‐α (ab6671; Abcam, Shanghai, China), anti‐IL‐1β (ab226918; Abcam) or anti‐IL‐6 (ab208113; Abcam) at 4°C overnight. The sections were then incubated in the secondary goat anti‐rabbit IgG for 30 min and were added to SABC for colour appearance. Finally, the haematoxylin was used for the counterstaining of the nucleus for 2 min.

According to previous reports (Horsfall, *et al.*, 1989; Dong, *et al.*, 2005; Brujan, *et al.*, 2009), quantitative analysis of immunoreactive staining was conducted by quantifying the integrated optical density (IOD), a parameter representing the expression levels of IL‐1β, IL‐6 or TNF‐α in inflamed uteri. We selected two researchers who were blinded to the treatment to select the areas of the slides independently. Briefly, three images were taken from each specimen under a magnification of 400×. The final IOD for each sample was measured by the mean IOD of immunoreactive staining area in three different images, subtracting the negative staining intensity using an Image‐Pro plus 6.0 software (MediaCybernetics, Inc., Silver Spring, MD, USA).

### Statistical analysis

All statistical analyses were performed with SPSS 17.0 (SPSS Inc, Chicago, IL, USA) . The data are expressed as the means ± SD. Comparisons between the *Trem‐1^−/−^* and WT groups after PBS or LPS treatment were performed using Student’s *t*‐test. *P* < 0.05 was considered statistically significant.

## Conflict of interest

None declared.

## Author contributions

LH and WKL designed the experiments. WKL, HMZ and ZLW conducted experiments. JGC and MXD provided support for image acquisition. HMZ and LH analyzed the results and wrote the manuscript.
